# Differential detection by breast density for digital breast tomosynthesis versus digital mammography population screening: a systematic review and meta-analysis

**DOI:** 10.1038/s41416-022-01790-x

**Published:** 2022-03-28

**Authors:** Tong Li, Nehmat Houssami, Naomi Noguchi, Aileen Zeng, M. Luke Marinovich

**Affiliations:** 1 The Daffodil Centre, The University of Sydney, a joint venture with Cancer Council New South Wales, Sydney, NSW Australia; 2 School of Public Health, Faculty of Medicine and Health, The University of Sydney, Sydney, NSW Australia; 3 Curtin School of Population Health, Curtin University, Bentley, WA Australia

**Keywords:** Cancer screening, Cancer imaging, Breast cancer

## Abstract

**Background:**

We examined whether digital breast tomosynthesis (DBT) detects differentially in high- or low-density screens.

**Methods:**

We searched six databases (2009–2020) for studies comparing DBT and digital mammography (DM), and reporting cancer detection rate (CDR) and/or recall rate by breast density. Meta-analysis was performed to pool incremental CDR and recall rate for DBT (versus DM) for high- and low-density (dichotomised based on BI-RADS) and within-study differences in incremental estimates between high- and low-density. Screening settings (European/US) were compared.

**Results:**

Pooled within-study difference in incremental CDR for high- versus low-density was 1.0/1000 screens (95% CI: 0.3, 1.6; *p* = 0.003). Estimates were not significantly different in US (0.6/1000; 95% CI: 0.0, 1.3; *p* = 0.05) and European (1.9/1000; 95% CI: 0.3, 3.5; *p* = 0.02) settings (*p* for subgroup difference = 0.15). For incremental recall rate, within-study differences between density subgroups differed by setting (*p* < 0.001). Pooled incremental recall was less in high- versus low-density screens (−0.9%; 95% CI: −1.4%, −0.4%; *p* < 0.001) in US screening, and greater (0.8%; 95% CI: 0.3%, 1.3%; *p* = 0.001) in European screening.

**Conclusions:**

DBT has differential incremental cancer detection and recall by breast density. Although incremental CDR is greater in high-density, a substantial proportion of additional cancers is likely to be detected in low-density screens. Our findings may assist screening programmes considering DBT for density-tailored screening.

## Background

Digital breast tomosynthesis (DBT) provides reconstructed, quasi-three-dimensional mammographic images of the breast, and has been proposed to improve cancer detection in screening through better visualisation of lesions that may be obscured by dense and/or overlapping breast tissue on conventional (two-dimensional) digital mammography (DM) [1]. In addition, by minimising cancer-mimicking artefacts associated with overlapping breast tissue, DBT may reduce high baseline rates of recall to further assessment [2]. Multiple studies have compared DBT and DM in breast cancer screening, including six published systematic reviews [3–8]. All of these reviews reported that detection measures favoured DBT (compared to DM) for breast cancer screening; however, none reported screening detection measures by high and low breast density. High mammographic density (having heterogeneously or extremely dense breasts [9]) is associated with an increased risk of breast cancer [10], including interval breast cancer [11]. Evidence on whether screening performance measures for DBT compared to DM differ by breast density is of interest to population breast cancer screening programmes, and could inform potential adoption of DBT screening in whole or subgroups of the population.

We address this critical knowledge gap by specifically examining cancer detection and recall rates for DBT versus DM by breast density, substantially extending our previous meta-analytic methods [4] to generate new evidence relevant to breast cancer screening practice. We therefore performed a systematic review and meta-analysis to determine if DBT screening is detecting differentially from DM screening in women with dense (high density) or non-dense breasts (low density).

## Methods

### Search strategy

This systematic review and meta-analysis followed the Preferred Reporting Items for Systematic Reviews and Meta-Analyses (PRISMA) guidelines [12]. As our previous work [4] (PROSPERO, CRD42016038998) conducted a systematic literature search from 2009 to July 2017, we updated the search strategy to perform a literature search from 1 January 2017 to 23 November 2020 in six Ovid electronic databases (EMBASE, PREMEDLINE, ACP Journal Club, Cochrane Controlled Trials Register, Cochrane Database of Systematic Reviews, and Database of Abstracts of Reviews of Effectiveness). The full search strategy is detailed in Supplementary Method 1.

### Eligibility criteria

Studies were eligible when they included asymptomatic women who attended population-based breast cancer screening programmes; compared DBT with DM; reported cancer detection and/or recall by breast density using American College of Radiology Breast Imaging Reporting and Database System (BI-RADS) [9] (any edition); and were reported in English. Detailed inclusion and exclusion criteria are available in Supplementary Method 2. Studies using either a paired design (i.e. all participants underwent DBT and DM, allowing within-participant comparison) or unpaired design (i.e. comparison of separate groups that underwent DBT, with or without DM, versus DM alone) were both eligible for inclusion. To ensure that density classification was consistent for the purpose of pooling estimates by density strata, we did not include studies using an automated density.

### Study selection

Titles and abstracts were screened by one author (TL) to determine whether studies met the eligibility criteria for full-text assessment and a sample of 25% was screened independently by another author (MLM) as a quality assurance process. The full-text assessment was conducted by one author (TL) with consultation from a second author (MLM) if required.

### Data extraction

Data extraction was performed by one author (TL), with another independent extraction by one of two other authors (NN and AZ). Any disagreements were resolved by discussion and consensus, or with arbitration by a third author (MLM) when needed.

The following data were extracted into an Excel spreadsheet using predefined cells: first author, publication date, country, study design, screening interval, years of participant enrolment, DBT views, DBT modality, DBT screening reading strategy, participants’ age (median or mean), and the number of participants and outcomes (cancers detected, recalls) per modality in each density category. Breast density information was extracted according to the BI-RADS density classification of a-d [9] (or 1–4 [13]) when available, and the combined categories of low density (BI-RADS a + b/1 + 2) and high density (BI-RADS c + d/3 + 4) when studies did not report the full BI-RADS classification. Because there were more studies reporting by combined (and less reporting by four) categories of density, we used the binary low- and high-density classification to standardise these data and allow statistical pooling across studies. This approach avoided excluding a substantial number of otherwise eligible studies.

### Quality assessment criteria

Quality assessment of all eligible studies was performed by one author (TL) in consultation with two other authors (MLM and NH) when required, using appraisal criteria adapted from QUADAS-2 [14]. Each study was assessed for risk of bias under four domains covering patient selection, index test, reference standard, and flow and timing. The first three domains were also assessed in terms of concerns regarding applicability.

### Statistical analysis

Study characteristics were summarised descriptively using median values and ranges. For both DBT and DM, estimates of cancer detection rate (CDR; per 1000 screens) and recall rate (percent) were calculated for low- and high-density strata within each study, and exact 95% confidence intervals (CIs) were computed. Summary estimates of CDR and recall rates for DM (baseline) and DBT were derived and compared between screening settings using PROC GLIMMIX with random effects for study in SAS 9.4 (SAS Institute, Cary, NC, US). Incremental estimates (risk differences), calculated as the study-level differences between modalities (DBT minus DM) in CDR and recall rate, were pooled separately for low- and high-density strata using the inverse variance method with random effects (DerSimoneon and Laird method as implemented in RevMan 5.4.1, The Cochrane Collaboration, 2020 [15]). Standard errors of the risk differences were calculated based on differences in two independent proportions for unpaired study designs. For paired study designs, PROC GENMOD in SAS was used to take account of the pairing of results within an individual when computing the standard error of the difference in proportions. These estimates were then input into RevMan for meta-analysis. Chi-squared tests of differences between the separate pooled estimates for density strata were not performed due to inappropriate standard errors (arising from the same studies contributing to both density strata) and the potential for bias [16, 17].

For the main analyses comparing density strata, we used PROC GENMOD (with the REPEATED statement for paired studies) to model the interaction between modality (DBT versus DM) and breast density (high versus low) for each study. Interaction terms (corresponding to the within-study difference between density strata in incremental CDR and recall rate) and their standard errors were input into RevMan and pooled using the inverse variance method with random effects [16, 17].

Analyses were stratified by screening setting (European versus US studies) based on a priori evidence of a difference in CDR and recall rate [4]. Differences between screening setting subgroups were assessed using the Chi-squared test. Sensitivity analyses were undertaken to include only studies that reported both CDR *and* recall rate to investigate the effect on pooled estimates. Heterogeneity was assessed using the *I*^2^ statistic with values >50% representing substantial or considerable heterogeneity [15].

Pooled estimates of incremental CDR and recall rate, and the within-study differences between density strata, were incorporated in an epidemiological model simulating plausible scenarios in population screening practice. Simplified decision trees (Supplementary Method 3) were used to apply conditional probabilities to a hypothetical screening population of 10,000 women where the screening setting and proportion of the population with low density were varied. Estimates of the proportion of the population with low density were derived from the median and range of study-specific values reported by European and US studies. For each screening setting and density subgroup, predictions of the number of additional cancers detected and additional women recalled by DBT per 10,000 screens were calculated by multiplying the total number in the population, the proportion of low (or high) density, and the relevant pooled incremental estimate derived from the meta-analysis. A detailed description of this modelled prediction can be found in Supplementary Method 3.

All tests of statistical significance were two-sided. The level chosen for statistical significance was 0.05.

## Results

### Study characteristics

An initial 565 studies were identified for title and abstract screening, of which 13 studies were eligible for inclusion (Supplementary Fig. 1) in our data synthesis and pooling [18–30], enrolling 1,238,735 participants/examinations (522,846 with DBT and 715,889 with DM) between 2010 and 2017. The study-level median age was 57.0 (range 54.5–59.0) years for DBT and 57.6 (range 53.8–58.6) years for DM cohorts, and the median proportion of women with low breast density was 63% (range 37%-83%) [18–30]. As summarised in Table 1, eight studies were based on US populations (using single reading) and five studies were Europe-based (using double-reading). European studies predominantly used a biennial screening interval, while US studies were assumed to employ mostly annual screening. Compared to US studies, European studies had significantly higher pooled CDR and lower pooled recall rate with DM (baseline) (Supplementary Tables 1 and 2; *p* < 0.05 for all comparisons between screening settings). These studies covered three modes of DBT, including DBT + DM (*n* = 11), DBT + SM (synthesised mammography) (*n* = 1), and DBT + DM + SM (*n* = 1), hereby referred to as DBT.

**Table 1:** Characteristics of the included studies.

Study^a^	Region	Design	Screening interval	Enrolment years	DBT mode	DBT reading strategy	DBT mean/median age	DM mean/median age	Breast density analysis level	Reported metrics
Bernardi et al. [18]	Europe	Prospective	Biennial	2013–2015	DBT + DM	Double^e^	58	58	Examinations	CDR and RR
Caumo et al. [19]	Europe	Prospective	Biennial	2014–2016	DBT + SM	Double^e^	59	58	Participants	CDR only
Ciatto et al. [20]	Europe	Prospective	Biennial	2011–2012	DBT + DM	Double^e^	58	58	Examinations	CDR and RR
Romero-Martín et al. [21]	Europe	Prospective	Biennial	2015–2016	DBT + DM + SM^d^	Double^e^	57.6	57.6	Examinations	CDR only
Zackrisson et al. [22]	Europe	Prospective	Biennial^b^	2010–2015	DBT + DM	Double^f^	57	57	Participants	CDR and RR
Alsheik et al. [23]	US	Retrospective	Annual^c^	2015–2017	DBT + DM	Single	57.8	58.6	Examinations	CDR and RR
Conant et al. [24]	US	Retrospective	Annual^c^	2011–2014	DBT + DM	Single	NR	NR	Examinations	CDR and RR
Friedewald et al. [25]	US	Retrospective	Annual^c^	2010–2012	DBT + DM	Single	56.2	57	Examinations	CDR and RR
Haas et al. [26]	US	Retrospective	Annual^c^	2011–2012	DBT + DM	Single	55.8	57.5	Participants	RR only
McCarthy et al. [27]	US	Retrospective	Annual^c^	2010–2013	DBT + DM	Single	56.7	56.9	Participants	CDR and RR
Rose et al. [28]	US	Retrospective	Annual^c^	2010–2012	DBT + DM	Single	54.5	53.8	Examinations	CDR and RR
Sharpe et al. [29]	US	Retrospective	Annual^c^	2011–2014	DBT + DM	Single	55.7	57.6	Examinations	RR only
Starikov et al. [30]	US	Retrospective	Annual^c^	2013–2013	DBT + DM	Single	NR	NR	Examinations	CDR and RR

*DBT* digital breast tomosynthesis, *DM* digital mammography, *SM* synthesised mammography, *CDR c*ancer detection rate, *RR* recall rate, *NR* not reported.

^a^All studies used two-view DBT except for Zackrisson et al. [22] which used one-view (mediolateral oblique) DBT.

^b^Biennial for women aged 55–74 years and 18 months for women aged 40–54 years.

^c^Not reported but the US studies are likely to be predominantly annual screening.

^d^Double reading of DBT + DM + SM and DBT + SM.

^e^Recall at either read.

^f^With consensus meeting.

### Risk of bias and applicability

Supplementary Fig. 2a–c provide a summary of the risk of bias and applicability concerns. Five of the 13 studies were rated as low risk of bias and low concerns of applicability [18–21, 27]. The main reason for a high or unclear risk of bias and/or applicability concerns was in the domain of patient selection due to reported [24, 26, 29, 30] or possible [23, 25, 28] selection bias from women with specific characteristics such as dense breasts being differentially referred to DBT or DM [24, 30], self-selection or referral [24, 28], equipment availability [26, 29], and/or hybrid settings of concurrent DBT and DM [23, 25]. Other reasons were related to flow and timing under risk of bias (e.g. examinations using non-standard density terms were excluded [29]) and index test under applicability concerns (e.g. one-view DBT [22]).

### Incremental cancer detection rate (CDR) for DBT by breast density

**Fig. 1:**
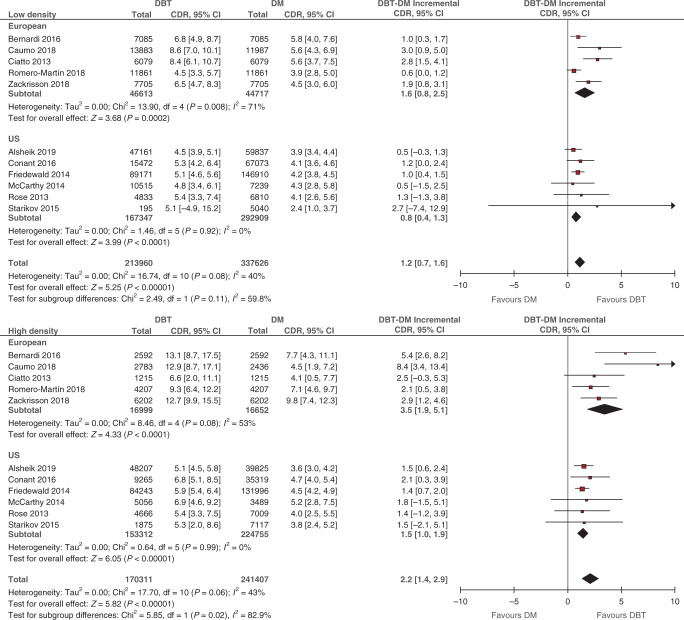
Difference in cancer detection rate (incremental CDR) between DBT and DM stratified by breast density. Breast density was classified as low (BI-RADS a + b) and high (BI-RADS c + d) (see Data extraction). Squares with horizontal lines represent individual study estimates and 95% CIs. Diamonds represent pooled estimates of incremental CDR for DBT over DM and 95% CIs. Additional data were supplied by study authors for Alsheik et al. [23]. CI confidence interval, df degrees of freedom.

Eleven studies reported on CDR, covering 384,271 participants/examinations undergoing DBT (213,960 in low and 170,311 in high density) and/or 579,033 participants/examinations undergoing DM (337,626 in low and 241,407 in high density). Supplementary Table 1 details study-specific number of screens, detected cancers and CDR for DBT and DM, stratified by breast density and screening setting. Pooled estimates of incremental CDR for DBT over DM, stratified by density and screening setting, are displayed in Fig. 1. DBT was associated with a statistically significant increase in CDR in both density strata. For European studies, the pooled incremental CDR in low density was 1.6 per 1000 screens (95% CI: 0.8, 2.5; *p* < 0.001; *I*^2^ = 71%) and the pooled incremental CDR in high density was 3.5 per 1000 screens (95% CI: 1.9, 5.1; *p* < 0.001; *I*^2^ = 53%). For US studies, the pooled incremental CDR in low density was 0.8 per 1000 screens (95% CI: 0.4, 1.3; *p* < 0.001; *I*^2^ = 0%) and the pooled incremental CDR in high density was 1.5 per 1000 screens (95% CI: 1.0, 1.9; *p* < 0.001; *I*^2^ = 0%).

**Fig. 2:**
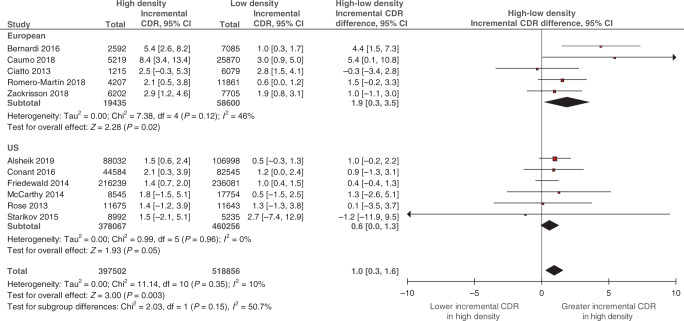
Difference between high- and low-density subgroups in DBT’s incremental CDR. Breast density was classified as low (BI-RADS a + b) and high (BI-RADS c + d) (see Data extraction). Squares with horizontal lines represent individual study estimates and 95% CIs. Diamonds represent pooled estimates in incremental CDR for high versus low density and 95% CIs. Additional data were supplied by study authors for Alsheik et al. [23]. CI confidence interval, df degrees of freedom.

Within-study differences between density strata were pooled in Fig. 2. For all studies combined, the pooled difference in incremental CDR for high versus low density was 1.0 per 1000 screens (95% CI: 0.3, 1.6; *p* = 0.003; *I*^2^ = 10%), indicating that the increase in CDR associated with DBT was statistically significantly greater in high compared with low density. When stratified by screening setting, a larger difference between density strata in incremental CDR was found for European studies (1.9 per 1000 screens; 95% CI: 0.3, 3.5; *p* = 0.02; *I*^2^ = 46%) compared to US studies (0.6 per 1000 screens; 95% CI: 0.0, 1.3; *p* = 0.05; *I*^2^ = 0%), but this subgroup difference was not statistically significant (*p* = 0.15).

In sensitivity analyses where only studies reporting both CDR *and* recall rate were included (*n* = 9), pooled estimates of incremental CDR (Supplementary Fig. 3) and the within-study difference between density strata (Supplementary Fig. 4) did not change substantially.

### Incremental recall rate for DBT by breast density

**Fig. 3:**
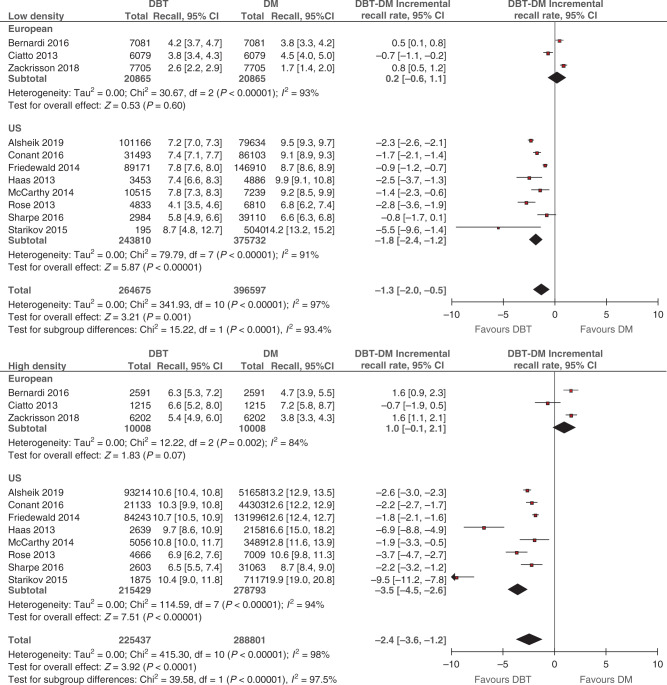
Difference in recall rate (incremental recall rate) between DBT and DM stratified by breast density. Breast density was classified as low (BI-RADS a + b) and high (BI-RADS c + d) (see Data extraction). Squares with horizontal lines represent individual study estimates and 95% CIs. Diamonds represent pooled estimates of incremental recall rate for DBT over DM and 95% CIs. Additional data were supplied by study authors for Alsheik et al. [23] and Zackrisson et al. [22]. CI confidence interval, df degrees of freedom.

Eleven studies reported recall data, including 490,112 participants/examinations undergoing DBT (264,675 in low and 225,437 and in high density) and/or 685,398 participants/examinations undergoing DM (396,597 in low and 288,801 in high density). Supplementary Table 2 details study-specific data for screen numbers, recalled cases and recall rates for DBT and DM, stratified by breast density and screening setting. Pooled estimates of incremental recall rate for DBT over DM, stratified by density and screening setting, are displayed in Fig. 3. For European studies, the pooled incremental recall rate was 0.2% (95% CI: −0.6%, 1.1%; *p* = 0.60; *I*^2^ = 93%) in low density and 1.0% (95% CI: −0.1%, 2.1%; *p* = 0.07; *I*^2^ = 84%) in high density. For US studies, DBT was associated with a statistically significant absolute decrease in recall rate of −1.8% (95% CI: −2.4%, −1.2%; *p* < 0.001; *I*^2^ = 91%) in low density and −3.5% (95% CI: −4.5%, −2.6%; *p* < 0.001; *I*^2^ = 94%) in high-density strata.

**Fig. 4:**
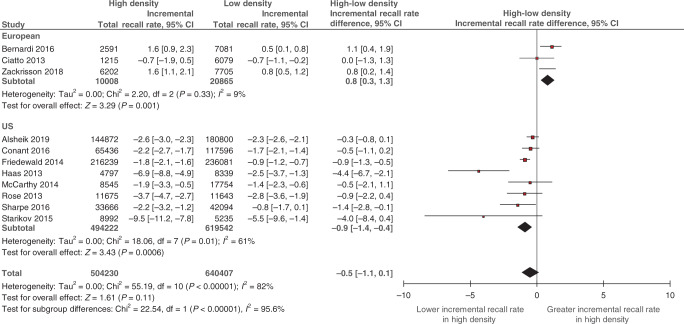
Difference between high- and low-density subgroups in DBT’s incremental recall rate. Breast density was classified as low (BI-RADS a + b) and high (BI-RADS c + d) (see Data extraction). Squares with horizontal lines represent individual study estimates and 95% CIs. Diamonds represent pooled estimates in incremental recall rate for high versus low and 95% CIs. Additional data were supplied by study authors for Alsheik et al. [23] and Zackrisson et al. [22]. CI confidence interval, df degrees of freedom.

The pooling of within-study differences between density strata is displayed in Fig. 4. Stratification by screening setting showed a statistically significant difference between US and European studies subgroups (*p* < 0.001). In European screening studies the incremental recall rate was statistically significantly *greater* in high versus low density (0.8%; 95% CI: 0.3%, 1.3%; *p* = 0.001; *I*^2^ = 9%). In contrast, for US screening studies, the incremental recall rate was statistically significantly *less* in high versus low density (−0.9%; 95% CI: −1.4%, −0.4%; *p* < 0.001; *I*^2^ = 61%).

In sensitivity analyses where only studies reporting both CDR *and* recall rate were included (*n* = 9), pooled estimates of incremental recall rate (Supplementary Fig. 5) and the within-study difference between density strata (Supplementary Fig. 6) did not change substantially.

### Modelled predictions of additional cancers detected and women recalled by DBT in population screening

Pooled estimates of incremental CDR and recall rate (Figs. 1 and 3), and the differences between density strata (Figs. 2 and 4), were applied to different scenarios defined by screening setting (US or European) and the percentage of low breast density in the screening population (‘median’, ‘maximum’ and ‘minimum’ percentage based on density distributions in the included studies; see Supplementary Method 3). Across all scenarios, the predicted total number of *additional* cancers detected by DBT ranged from 9 to 25 per 10,000 screens (Table 2). The ratio of the numbers of additional cancers detected in high versus low density depended on the percentage of screens with low density. Despite evidence of greater incremental CDR in high density (Fig. 2), the number of additional cancers detected by DBT in women with low density exceeded the number in high density for the ‘maximum’ percentage of low-density screens. The reverse was apparent for the ‘minimum’ percentage estimates. These patterns were observed regardless of the screening setting.

**Table 2:** Modelled predictions of the number of additional cancers detected and cases recalled by DBT in a cohort of 10,000 screens.

Screening setting	Percentage of screens with low density^a^	Additional cancer detection in a cohort of 10,000 screens						
		Women with low breast density			Women with high breast density			All women
		Number of women	DBT-DM incremental CDR (per 1000 screens)^b^	Predicted number of additional cancers detected by DBT^c^	Number of women	DBT-DM incremental CDR (per 1000 screens)^d^	Predicted number of additional cancers detected by DBT^c^	Total predicted number of additional cancers detected by DBT per 10,000 screens^e^
European	Median (74%)	7400	1.6	12	2600	3.5	9	21
	Maximum (83%)	8300	1.6	13	1700	3.5	6	19
	Minimum (55%)	5500	1.6	9	4500	3.5	16	25
US	Median (55%)	5500	0.8	4	4500	1.4	6	10
	Maximum (68%)	6800	0.8	5	3200	1.4	4	9
	Minimum (37%)	3700	0.8	3	6300	1.4	9	12

Screening setting	Percentage of screens with low density^a^	Additional recalls in a cohort of 10,000 screens						
		Women with low breast density			Women with high breast density			All women
		Number of women	DBT-DM incremental recall rate (%)^b^	Predicted number of additional cases recalled by DBT^c^	Number of women	DBT-DM incremental recall rate (%)^d^	Predicted number of additional cases recalled by DBT^c^	Total predicted number of additional cases recalled by DBT per 10,000 screens^e^
European	Median (74%)	7400	0.2	15	2600	1.0	26	41
	Maximum (83%)	8300	0.2	17	1700	1.0	17	34
	Minimum (55%)	5500	0.2	11	4500	1.0	45	56
US	Median (55%)	5500	−1.8	−99	4500	−2.7	−121	−220
	Maximum (68%)	6800	−1.8	−122	3200	−2.7	−86	−208
	Minimum (37%)	3700	−1.8	−67	6300	−2.7	−170	−237

^a^Refers to median, maximum and minimum study-level percentage of women with low breast density derived from European studies (*N* = 5) and US studies (*N* = 8) (Supplementary Method 3).

^b^Pooled estimates for low-density screens by screening setting subgroup from Fig. 1 (CDR) or Fig. 3 (recall).

^c^Number of women in density subgroup multiplied by incremental CDR or recall rate.

^d^Pooled incremental estimate for high versus low density from Fig. 2 (CDR) or Fig. 4 (recall) summed with a pooled estimate for low-density screens (Supplementary Method 3).

^e^Sum of additional cancers detected or cases recalled in both low- and high-density subgroups.

The estimated number of *additional* women recalled by DBT ranged from −237 to 56 per 10,000 screens (Table 2). For European screening, DBT was associated with a relatively small increase in the number of recalls. At the ‘maximum’ percentage of low-density screens, the number of additional recalls was equal in the high- and low-density groups, but at lower percentages, the number of recalls was greater in high compared with low density. For US screening, the ratio of additional recalls in high versus low density reflected the pattern observed for CDR. At the ‘maximum’ percentage of low-density screens, the reduction in the number of women recalled was greater in the low-density than in the high-density group (and vice versa at the ‘minimum’ percentage).

## Discussion

The adoption of DBT in place of DM for population breast cancer screening has progressed rapidly, particularly in the US, whereas elsewhere there is conditional approval or restricted use of DBT in screening programmes [31]. Some population-based screening programmes do not currently endorse using DBT instead of DM but encourage its evaluation in prospective trials [32, 33]. Mammographic breast density, a long-established independent risk factor for breast cancer, has gained increased attention since the introduction of breast density legislation in the US [34, 35], and there is a suggestion that DBT may be more effective for screening women with dense breasts [4, 36]. In this systematic review, we focused on estimating changes in cancer detection and recall associated with screening by DBT versus DM according to breast density. Our meta-analysis provides evidence that DBT increases cancer detection in both low- and high-density screening examinations regardless of the screening setting. Importantly, we also show that DBT has differential incremental detection (versus DM) by breast density, meaning that the increase in CDR is greater in high (versus low) density screens. Conversely, both the incremental recall rate for DBT and the differential incremental recall by density varied by screening setting.

Our estimates provide new synthesised evidence on the performance of DBT, noting that other systematic reviews [3–8] have not investigated the differential performance of DBT by density. One other review reported detection for DBT versus DM solely in screens classified as dense [37]. Our work showed that DBT detected more cancers than DM in both low- and high-density screens, and that DBT substantially improved CDR in high-density compared to low-density screens (pooled difference in incremental CDR 1.0 per 1000 screens). This improvement was more evident in studies undertaken in Europe (1.9 per 1000 screens) than in US studies (0.6 per 1000 screens). Although the difference between screening settings was not statistically significant, pooling within-study interactions is likely to have low power to detect such subgroup differences [16]. A greater contribution by DBT to cancer detection in European screening practice is likely to reflect a longer time interval between screens, however other differences between European and US screening practices (e.g. double versus single-reading) may also contribute to this difference.

The pooled difference in the incremental recall rate between low- and high-density screens differed between the screening settings. For European screening studies, a greater *increase* in DBT’s incremental recall was observed in high compared with low-density screens (pooled absolute difference in recall rate 0.8%) with little heterogeneity (*I*^2^ = 9%). In contrast, for US screening studies, there was a greater *decrease* in recall for DBT in high- than in low-density screens (pooled absolute difference in recall rate −0.9%). Although there was substantial heterogeneity in the magnitude of this estimate (*I*^2^ = 61%), all US screening studies were consistent in the direction of the difference (Fig. 4). The opposing directions of the estimates from European and US studies are likely because the ‘baseline’ recall rates for DM in US screening studies were larger than those reported in European screening studies (Supplementary Table 2). Our results suggest that DBT has a beneficial effect in reducing recalls in women with dense breasts in US screening practice but may lead to increased recall in high-density screens in European screening programmes.

Our estimates of DBT’s differential incremental detection and recall (versus DM) by breast density are relevant to screening programmes worldwide contemplating whether DBT should be used for population screening, if such decisions were to be based on conventional screening measures. The data provided in Table 2, for example, showing the additional detection (or effect on recall) if DBT replaced DM screening, according to the observed percentage of breast density and screening setting, can inform plans for trials or implementation studies. A screening programme targeting women aged 50-years-old and above with a large proportion of participants with low-density breasts (as would be expected in many European programmes, and Australia’s programme [38]) would improve CDR overall through more detection in both low and high-density screens. In that setting, limiting DBT to those with high density would not achieve optimal outcomes from DBT screening. In contrast, if a European screening programme comprised a large proportion of participants with high breast density, much of the incremental CDR would be achieved by offering DBT to women with dense breasts. Our results may also be relevant to planning new research in risk-tailored screening [39].

There are limitations to this work that should be considered when using our findings. The included studies reported initial detection measures and lacked data on long-term health outcomes from DBT screening. It is therefore unknown whether DBT’s incremental detection will lead to incremental screening benefits by reducing breast cancer mortality. Also, most of the data reported on prevalent (initial) DBT screening, even though repeat breast screening represents the majority of screens in screening programmes. Therefore, it is possible our results may be less generalisable to repeat (incident) DBT screening. Another limitation is that we included studies that assessed breast density using BI-RADS density classification, but excluded studies using automated assessment for consistency in meta-analysis. Given that automated density measures have only been recently introduced into practice [40–43], automated density should be assessed in future meta-analyses as the evidence develops. These issues reflect the still-evolving evidence base for DBT, a limitation inherent in evaluations of new health technologies that aim to inform implementation before practice becomes established and therefore more challenging to modify [44].

In addition, we have used ‘US screening’ and ‘European screening’ to classify studies, but this classification is only broadly indicative of screening practice—we acknowledge that varying practices exist in an inter-screen time interval and screen-reading strategy. For example, US studies may not have performed all screening annually, and other factors that differ between US and European studies, such as single versus double-reading and the generally high recall rates in US studies, may account for some of the observed differences in incremental CDR and recall rates.

Internationally, the majority of population breast cancer screening programmes use DM, but many are contemplating the potential role of DBT screening. This is occurring in an evolving population screening landscape that includes deliberation regarding density notification, and risk-tailored breast screening. Our meta-analysis provides timely comparative estimates for DBT and DM screening showing that DBT has differential incremental cancer detection and recall by breast density. Therefore, our synthesised evidence may assist screening policy, planning of research and individual screening recommendations.

## Supplementary information

Supplementary Information

PRISMA checklist

A reproducibility checklist

## Data Availability

All data generated or analysed during this study are included in this published article and its Supplementary Information files.
