# Supplementary material for: Differential detection by breast density for digital breast tomosynthesis versus digital mammography population screening: a systematic review and meta-analysis
Source: Br J Cancer. 2022 Mar 28;127(1):116–25. doi: 10.1038/s41416-022-01790-x (PMC9276736; doi:10.1038/s41416-022-01790-x)
Supplement: Supplementary file 1 — Supplementary Information [file 41416_2022_1790_MOESM1_ESM.docx]

Supplementary Information

[Supplementary method 1: Search strategy 2](#_Toc92369000)

[Supplementary method 2: Inclusion and exclusion criteria 3](#_Toc92369001)

[Supplementary methods 3: Modelled predictions of number of additional cancers detected and cases recalled by DBT in a cohort of 10,000 screens 4](#_Toc92369002)

[Supplementary figure 1: PRISMA flowchart 6](#_Toc92369003)

[Supplementary figure 2a: Risk of bias and applicability concerns summary: review authors' judgements about each domain for each included study 7](#_Toc92369004)

[Supplementary figure 2b: Risk of bias and applicability concerns graph: review authors' judgements about each domain presented as percentages across included studies 8](#_Toc92369005)

[Supplementary figure 2c: Risk of bias and applicability concerns summary: review authors' judgements of each signaling question under each domain within risk of bias (in Sentence case) and concerns regarding applicability questions for the first three domains (in UPPERCASE) for each included study 9](#_Toc92369006)

[Supplementary table 1: Numbers of screens and detected cancers, and cancer detection rates for low and high density separately for DBT and DM, and incremental cancer detection rate for DBT versus DM 10](#_Toc92369007)

[Supplementary figure 3: Sensitivity analysis for difference in cancer detection rate (incremental CDR) between DBT and DM stratified by breast density (including studies reporting recall rate in addition to CDR) 11](#_Toc92369008)

[Supplementary figure 4: Sensitivity analysis for difference between high and low density subgroups in DBT’s incremental CDR (including studies reporting recall rate in addition to CDR) 12](#_Toc92369009)

[Supplementary table 2: Numbers of screens and recalled cases, and recall rates for low and high density separately for DBT and DM, and incremental recall rate for DBT versus DM 13](#_Toc92369010)

[Supplementary figure 5: Sensitivity analysis for difference in recall rate (incremental recall rate) between DBT and DM stratified by breast density (including studies reporting CDR in addition to recall rate) 14](#_Toc92369011)

[Supplementary figure 6: Sensitivity analysis for difference between high and low density subgroups in DBT’s incremental recall rate (including studies reporting CDR in addition to recall rate) 15](#_Toc92369012)

## Supplementary method 1: Search strategy

Search terms for EMBASE, PREMEDLINE, ACP Journal Club (ACP), Cochrane Controlled Trials Register (CCTR), Cochrane Database of Systematic Reviews (CDSR), and Database of Abstracts of Reviews of Effectiveness (DARE).

1. tomosynthesis.mp

2. dbt.mp

3. 3d mammogra*.mp

4. exp digital breast tomosynthesis/

5. or/1‐4

6. breast.mp

7. exp breast cancer/

8. exp breast/

9. or/6‐8

10. screening.mp

11. exp screening/

12. exp early cancer diagnosis/

13. or/10‐12

14. 5 and 9 and 13

15. limit 14 to yr="2009 ‐Current"

## Supplementary method 2: Inclusion and exclusion criteria

1 Participants: Asymptomatic women who attend population-based breast cancer screening program

- Non‐breast cancer indications will be excluded.
- Studies reporting on male breast cancer will be excluded.
- Studies mixing asymptomatic and symptomatic/diagnostic women will be excluded.

2 Exposure modality: Digital breast tomosynthesis (DBT)

- Conducted in population-based screening setting (i.e. not high risk screening, or preferentially recruiting women at increased risk or underserved women).
- Modalities include DBT alone, DBT+DM (digital mammography), DBT+SM (synthesised mammography), and DBT+DM+SM.
- Assessment/work-up, diagnosis, staging, subtype, surveillance and response assessment excluded.

3 Comparator modality: Conventional mammography

- Studies comparing tomosynthesis with alternative tests (i.e. not digital mammography), or with no comparator, will be excluded.

4 Outcomes

- Studies must report on cancer detection by density or recall by density using ACR BI-RADS classification.
- Studies reporting none of those outcomes will be excluded.
- Studies reporting on cancer detection/recall by density together with other factors will be excluded.
- Studies reporting density using percentage or volumetric density only will be excluded.

5 Study design

- Population-based screening studies
- Studies will be excluded if:

1. A non‐clinical study (e.g. case report, review, animal study, phantom study, study protocol, survey, letter/ communications, editorial, guideline, consensus statement, technical note, perspectives, viewpoints, corrections/errata, practice bulletins, etc.).
2. Mammographic/standardised image sets (multi‐reader or observer studies, not real‐life screening, enriched with cases, not representative of screening practice etc.).
3. Reported only as abstract.
4. Clinical trial register entry only.

6 Language

- Non‐English language studies will be excluded.

7 Superseded publications

- In cases of multiple publications based on the same study population, the most recent publication or the publication with the longest follow‐up (or reporting the required end‐points most clearly) will be included.
- Where multiple publications based on the same study population report different or complementary outcomes, relevant data will be extracted from those publications, but will be treated in the analysis as a single study.

## Supplementary methods 3: Modelled predictions of number of additional cancers detected and cases recalled by DBT in a cohort of 10,000 screens

Table 2 in the manuscript presents a basic epidemiological model that simulates representative scenarios for applying DBT in population breast cancer screening. Each row of table 2 may be described by a simplified decision tree applying conditional probabilities to a hypothetical screening population (N=10,000 screens). The first *chance node* of the tree represents the proportion of the screening population with high and low breast density. The relevant pooled estimates of incremental CDR or recall rate for DBT are then applied to each density branch of the decision tree^[[1]](#footnote-1)^. Finally, the estimates are multiplied to derive the predicted number of additional cancers detected or recalls by DBT in each density group (*end nodes*).

The example decision tree below (Figure SM3-1) describes the first row of Table 2, where Panel A * Panel B * Panel C = Panel D. For example, the predicted number of additional cancers detected by DBT in the low-density subgroup is 10,000 * 0.74 * 0.0016 = 12. The total number of additional cancers detected by DBT in the population can therefore be derived by summing the predicted number of additional cancers in each density subgroup (12 + 9 = 21 per 10,000 screens).


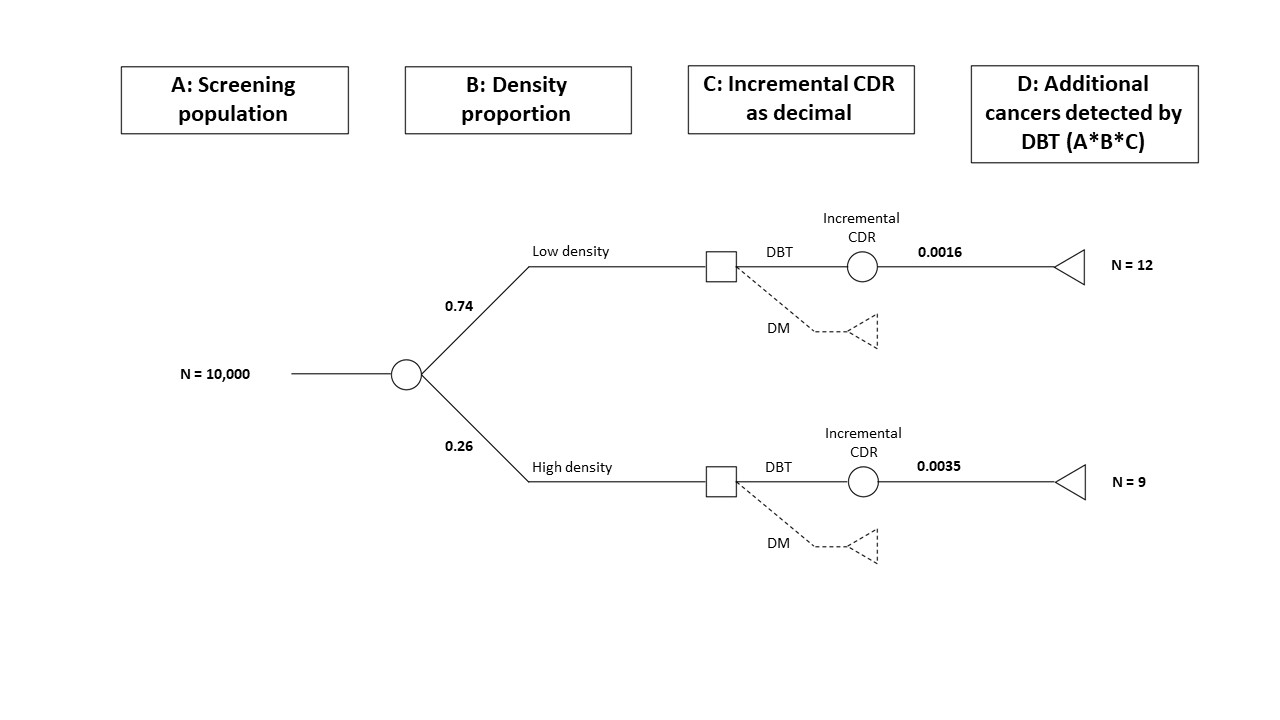


**Figure SM3-1: Simplified decision tree for the calculation of additional cancers by DBT in European screening settings (median percentage of screens with low breast density)**

By varying the density proportions and pooled incremental CDR and recall rate estimates, table 2 reports a two-way sensitivity analysis of the decision tree model. The data used to populate the decision tree are derived from evidence summarised in the systematic review:

1. The percentages of screens with low versus high density for each screening setting (Panel B) are informed by the distributions observed in the included studies (presented in Table SM3-1, below).
2. The incremental estimates for the low-density subgroup in each screening setting (Panel C top density branch) are derived from the meta-analyses presented in Figure 1 (CDR) and Figure 3 (recall rate).
3. The incremental estimates for the high-density subgroup (Panel C bottom density branch) are derived by summing i) the low-density estimates, and ii) the pooled within-study differences between high and low density from the meta-analyses presented in Figure 2 (CDR) and Figure 4 (recall rate).

The method for calculating incremental estimates for the high-density subgroup in (3) uses the methodologically appropriate estimate of the difference between density subgroups (i.e. pooled within-study differences), applied to a reference value (i.e. pooled estimate for low density). These calculated estimates may differ slightly from the pooled estimates for high density in Figures 1 and 3. Including the high density pooled estimates from Figures 1 and 3 in the decision trees does not change interpretation presented in the Results or the conclusions presented in the Discussion; nor does deriving an incremental estimate for the low-density subgroup by applying pooled within-study differences between density groups to the pooled estimate for high density as the reference value (data not shown).

| **Table SM3-1: Percentage of screens/women with low and high density by screening setting** | | |
| --- | --- | --- |
|  | **Low density** | **High density** |
| **European** | | |
| Ciatto 2013 | 83.3% | 16.7% |
| Caumo 2018 | 83.2% | 16.8% |
| Romero-Martín 2018 | 73.8% | 26.2% |
| Bernardi 2016 | 73.2% | 26.8% |
| Zackrisson 2018 | 55.4% | 44.6% |
| ***Median*** | ***74%*** | ***26%*** |
| ***Maximum*** | ***83%*** | ***45%*** |
| ***Minimum*** | ***55%*** | ***17%*** |
| **US** | | |
| McCarthy 2014 | 67.5% | 32.5% |
| Conant 2016 | 64.9% | 35.1% |
| Haas 2013 | 63.5% | 36.5% |
| Sharpe 2016 | 55.6% | 44.4% |
| Alsheik 2019 | 54.9% | 45.1% |
| Friedewald 2014 | 52.2% | 47.8% |
| Rose 2013 | 49.9% | 50.1% |
| Starikov 2015 | 36.8% | 63.2% |
| ***Median*** | ***55%*** | ***45%*** |
| ***Maximum*** | ***68%*** | ***63%*** |
| ***Minimum*** | ***37%*** | ***32%*** |

## Supplementary figure 1: PRISMA flowchart

Additional records identified from the previous systematic review
(n = 11)

Titles and abstracts screened (n =565)
(n=)

Eligible papers included in data synthesis
(n = 13)

Full text assessed
(n = 54)

Records after duplicates removed
(n = 565)

Records identified through database searching (2017- 23 November 2020)
(n = 633)

Identification

Eligibility

Screening

Excluded from screening of titles and abstracts with reasons (n = 511)

1 – wrong participants (n = 73)

2 – wrong exposure modality (n = 55)

3 – wrong comparator (n = 8)

4 – wrong outcomes (n = 18)

5 – wrong study design (n = 344)

6 – not in English (n = 11)

7 – superseded publications (n = 2)

Excluded from assessment of full text with reasons

(n =41)

1 – wrong participants (n = 0)

2 – wrong exposure modality (n = 2)

4 – wrong outcomes (n = 34)

5 – wrong study design (n = 0)

6 – not in English (n = 0)

7 – superseded publications (n = 4)

## Supplementary figure 2a: Risk of bias and applicability concerns summary: review authors' judgements about each domain for each included study

## Supplementary figure 2b: Risk of bias and applicability concerns graph: review authors' judgements about each domain presented as percentages across included studies

## Supplementary figure 2c: Risk of bias and applicability concerns summary: review authors' judgements of each signaling question under each domain within risk of bias (in Sentence case) and concerns regarding applicability questions for the first three domains (in UPPERCASE) for each included study


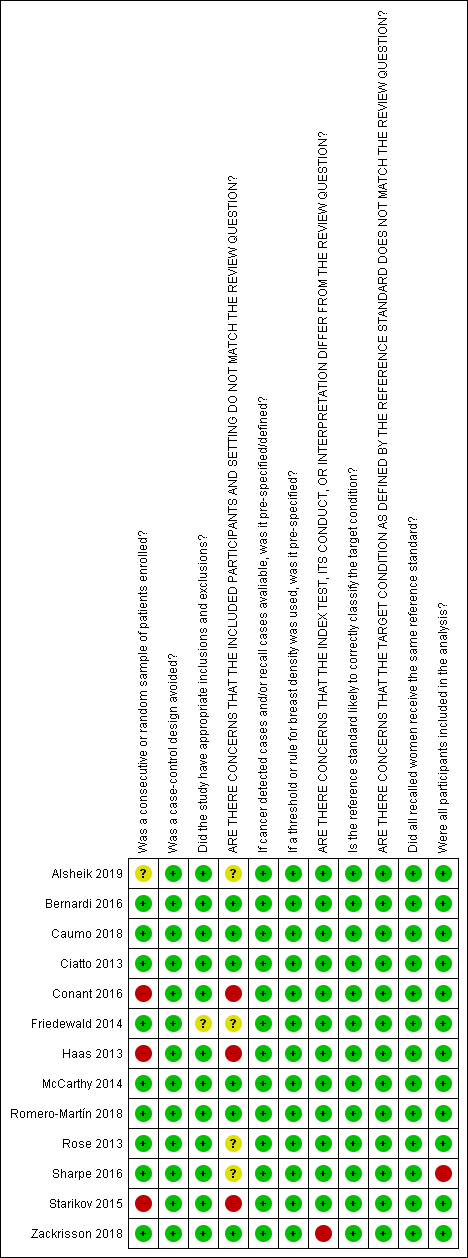


| Supplementary table 1: Numbers of screens and detected cancers, and cancer detection rates for low and high density separately for DBT and DM, and incremental cancer detection rate for DBT versus DM | | | | | | | | | | | | | | | | |
| --- | --- | --- | --- | --- | --- | --- | --- | --- | --- | --- | --- | --- | --- | --- | --- | --- |
| **Study** | **DBT** | | | | | | **DM** | | | | | | **Difference (DBT minus DM)** | | | |
|  | **Low density** | | | **High density** | | | **Low density** | | | **High density** | | | **Low density** | | **High density** | |
|  | **Screens, N** | **Cancers, N** | **CDR, per 1,000)**  **(95% CI)** | **Screens, N** | **Cancers, N** | **CDR, per 1,000)**  **(95% CI)** | **Screens, N** | **Cancers, N** | **CDR, per 1,000)**  **(95% CI)** | **Screens, N** | **Cancers, N** | **CDR, per 1,000)**  **(95% CI)** | **Incremental CDR, per 1,000 (95%CI)** | **P** | **Incremental CDR, per 1,000 (95%CI)** | **P** |
| **European studies** | | | | | | | | | | | | | | | | |
| Bernardi 2016 | 7085 | 48 | 6.8  (4.9 to 8.7) | 2592 | 34 | 13.1  (8.7 to 17.5) | 7085 | 41 | 5.8  (4.0 to 7.6) | 2592 | 20 | 7.7  (4.3 to 11.1) | 1.0  (0.3 to 1.7) | 0.008 | 5.4  (2.6 to 8.2) | <0.002 |
| Caumo 2018 | 13883 | 119 | 8.6  (7.0 to 10.1) | 2783 | 36 | 12.9  (8.7 to 17.1) | 11987 | 67 | 5.6  (4.3 to 6.9) | 2436 | 11 | 4.5  (1.9 to 7.2) | 3.0  (0.9 to 5.0) | 0.004 | 8.4  (3.4 to 13.4) | 0.001 |
| Ciatto 2013 | 6079 | 51 | 8.4  (6.1 to 10.7) | 1215 | 8 | 6.6  (2.0 to 11.1) | 6079 | 34 | 5.6  (3.7 to 7.5) | 1215 | 5 | 4.1  (0.5 to 7.7) | 2.8  (1.5 to 4.1) | <0.001 | 2.5  (-0.3 to 5.3) | 0.083 |
| Romero-Martín 2018 | 11861 | 53 | 4.5  (3.3 to 5.7) | 4207 | 39 | 9.3  (6.4 to 12.2) | 11861 | 46 | 3.9  (2.8 to 5.0) | 4207 | 30 | 7.1  (4.6 to 9.7) | 0.6  (0.0 to 1.2) | 0.071 | 2.1  (0.5 to 3.8) | 0.013 |
| Zackrisson 2018 | 7705 | 50 | 6.5  (4.7 to 8.3) | 6202 | 79 | 12.7  (9.9 to 15.5) | 7705 | 35 | 4.5  (3.0 to 6.0) | 6202 | 61 | 9.8  (7.4 to 12.3) | 1.9  (0.8 to 3.1) | 0.001 | 2.9  (1.2 to 4.6) | 0.001 |
| Summary estimate | 46613 | 321 | 6.8  (5.6 to 8.3) | 16999 | 196 | 11.3  (9.4 to 13.6) | 44717 | 223 | 5.0  (4.2 to 5.7) | 16652 | 127 | 7.3  (5.7 to 9.3) | 1.6  (0.8 to 2.5) | <0.001 | 3.5  (1.9 to 5.1) | <0.001 |
| **US studies** | | | | | | | | | | | | | | | | |
| Alsheik 2019* | 47161 | 210 | 4.5  (3.9 to 5.1) | 48207 | 247 | 5.1  (4.5 to 5.8) | 59837 | 235 | 3.9  (3.4 to 4.4) | 39825 | 144 | 3.6  (3.0 to 4.2) | 0.5  (-0.3 to 1.3) | 0.188 | 1.5  (0.6 to 2.4) | <0.001 |
| Conant 2016 | 15472 | 82 | 5.3  (4.2 to 6.4) | 9265 | 63 | 6.8  (5.1 to 8.5) | 67073 | 275 | 4.1  (3.6 to 4.6) | 35319 | 166 | 4.7  (4.0 to 5.4) | 1.2  (0.0 to 2.4) | 0.058 | 2.1  (0.3 to 3.9) | 0.024 |
| Friedewald 2014 | 89171 | 455 | 5.1  (4.6 to 5.6) | 84243 | 495 | 5.9  (5.4 to 6.4) | 146910 | 610 | 4.2  (3.8 to 4.5) | 131996 | 597 | 4.5  (4.2 to 4.9) | 1.0  (0.4 to 1.5) | 0.001 | 1.4  (0.7 to 2.0) | <0.001 |
| McCarthy 2014 | 10515 | 50 | 4.8  (3.4 to 6.1) | 5056 | 35 | 6.9  (4.6 to 9.2) | 7239 | 31 | 4.3  (2.8 to 5.8) | 3489 | 18 | 5.2  (2.8 to 7.5) | 0.5  (-1.5 to 2.5) | 0.643 | 1.8  (-1.5 to 5.1) | 0.295 |
| Rose 2013 | 4833 | 26 | 5.4  (3.3 to 7.4) | 4666 | 25 | 5.4  (3.3 to 7.5) | 6810 | 28 | 4.1  (2.6 to 5.6) | 7009 | 28 | 4.0  (2.5 to 5.5) | 1.3  (-1.3 to 3.8) | 0.332 | 1.4  (-1.2 to 3.9) | 0.297 |
| Starikov 2015 | 195 | 1 | 5.1  (-4.9 to 15.2) | 1875 | 10 | 5.3  (2.0 to 8.6) | 5040 | 12 | 2.4  (1.0 to 3.7) | 7117 | 27 | 3.8  (2.4 to 5.2) | 2.7  (-7.4 to 12.9) | 0.595 | 1.5  (-2.1 to 5.1) | 0.401 |
| Summary estimate | 167347 | 824 | 4.9  (4.1 to 5.9) | 153312 | 875 | 5.7  (5.1 to 6.5) | 292909 | 1191 | 4.1  (3.8 to 4.3) | 224755 | 980 | 4.2  (3.6 to 5.0) | 0.8  (0.4 to 1.3) | <0.001 | 1.5  (1.0 to 1.9) | <0.001 |
| DBT = Digital breast tomosynthesis; DM = Digital mammography; CDR = Cancer detection rate; CI = Confidential interval.  * Study authors provided additional data | | | | | | | | | | | | | | | | |

Supplementary figure 3: Sensitivity analysis for difference in cancer detection rate (incremental CDR) between DBT and DM stratified by breast density (including studies reporting recall rate in addition to CDR)
Breast density was classified as low (BI-RADS a+b) and high (BI-RADS c+d) (see Data extraction). Risk difference is incremental CDR (DBT-DM), expressed in decimal form. Squares with horizontal lines represent individual study estimates and 95% CIs. Diamonds represent pooled estimates of incremental CDR for DBT over DM and 95% CIs. Additional data were supplied by study authors for Alsheik et al 2019. CI = confidence interval; df = degrees of freedom; IV = inverse variance.


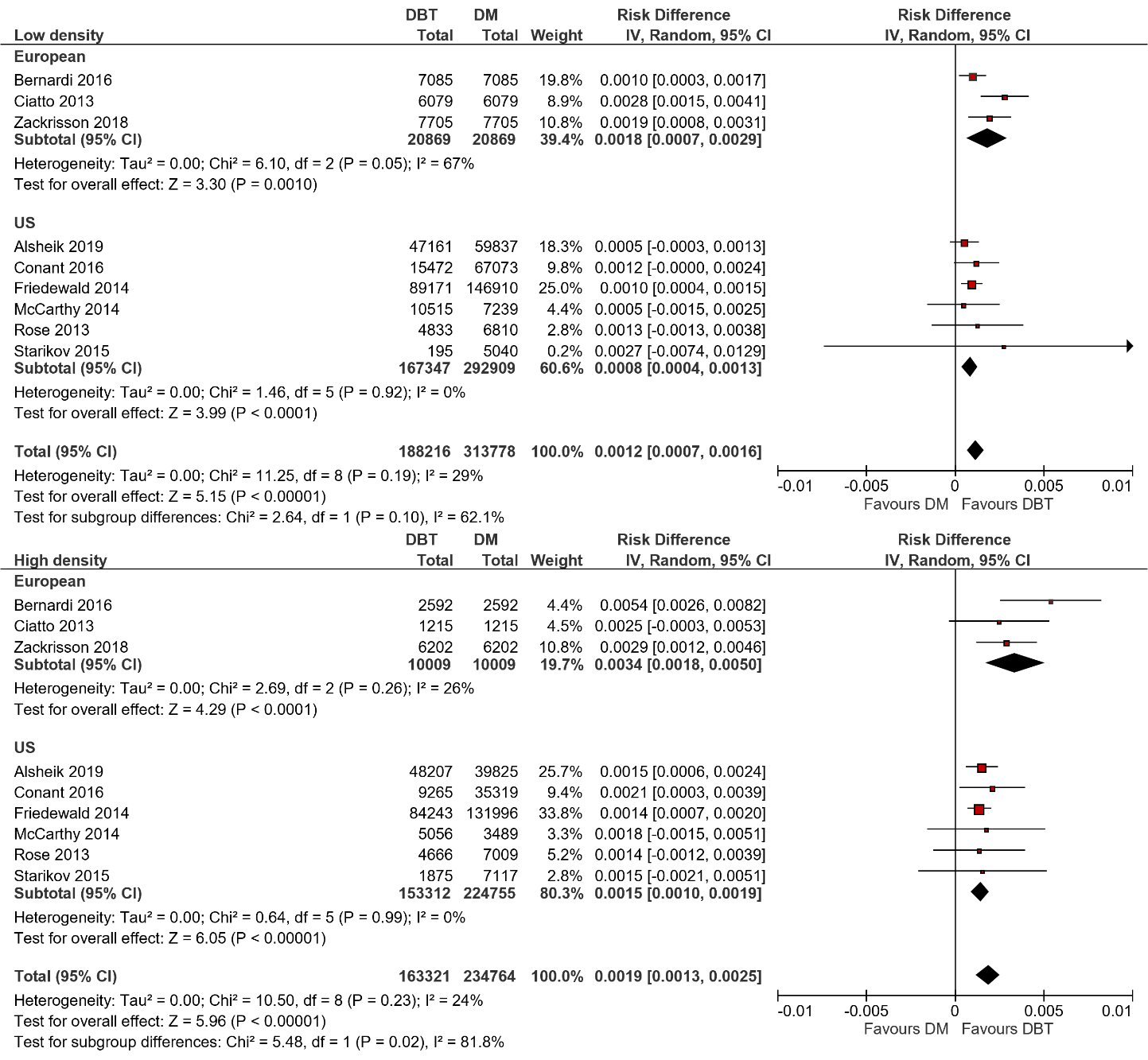


Supplementary figure 4: Sensitivity analysis for difference between high and low density subgroups in DBT’s incremental CDR (including studies reporting recall rate in addition to CDR)
Breast density was classified as low (BI-RADS a+b) and high (BI-RADS c+d) (see Data extraction). Risk difference is incremental CDR difference (high-low density), expressed in decimal form. Squares with horizontal lines represent individual study estimates and 95% CIs. Diamonds represent pooled estimates in incremental CDR for high versus low density and 95% CIs. Additional data were supplied by study authors for Alsheik et al 2019. CI = confidence interval; df = degrees of freedom; IV = inverse variance.


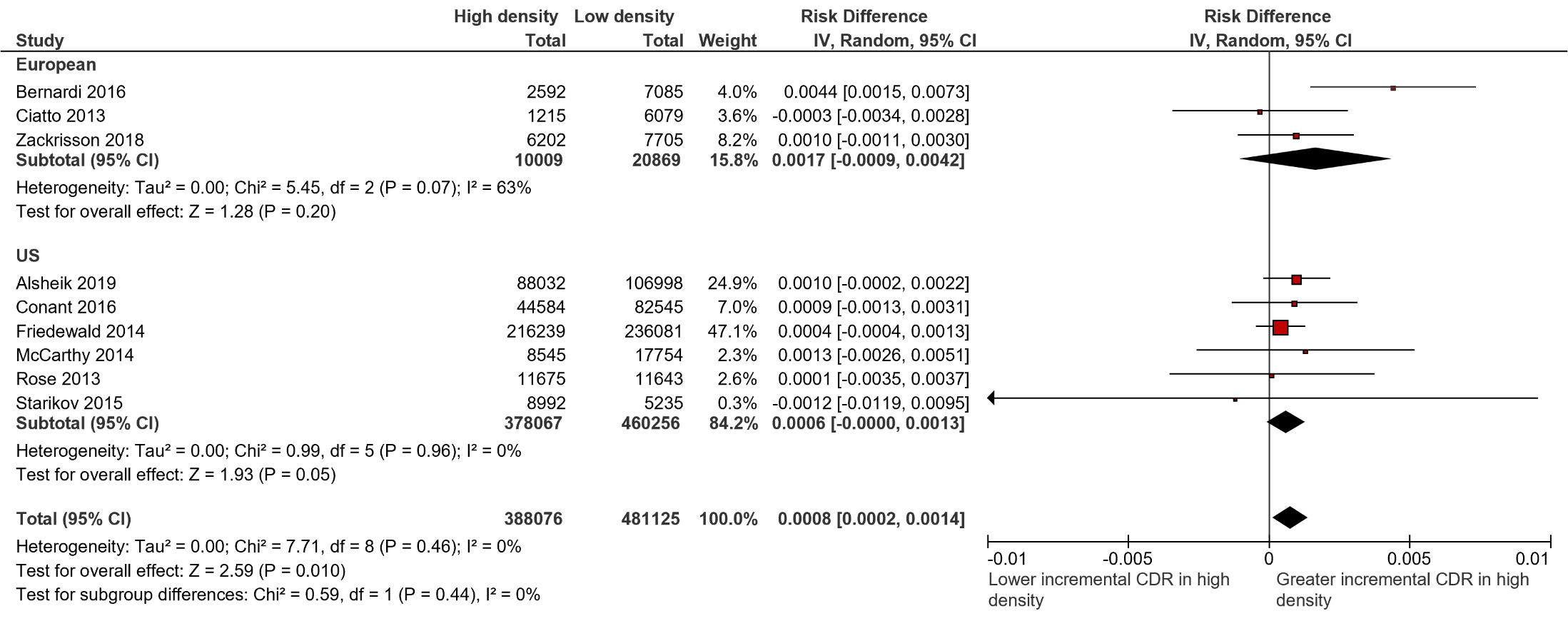


| Supplementary table 2: Numbers of screens and recalled cases, and recall rates for low and high density separately for DBT and DM, and incremental recall rate for DBT versus DM | | | | | | | | | | | | | | | | |
| --- | --- | --- | --- | --- | --- | --- | --- | --- | --- | --- | --- | --- | --- | --- | --- | --- |
| **Study** | **DBT** | | | | | | **DM** | | | | | | **Difference (DBT minutes DM)** | | | |
|  | **Low density** | | | **High density** | | | **Low density** | | | **High density** | | | **Low density** | | **High density** | |
|  | **Screens, N** | **Recalls, N** | **recall rate, % (95% CI)** | **Screens, N** | **Recalls, N** | **recall rate, % (95% CI)** | **Screens, N** | **Recalls, N** | **recall rate, % (95% CI)** | **Screens, N** | **Recalls, N** | **recall rate, % (95% CI)** | **Incremental recall rate discrepancy, % (95% CI)** | **P** | **Incremental recall rate discrepancy, %)**  **(95% CI)** | **P** |
| **European studies** | | | | | | | | | | | | | | | | |
| Bernardi 2016 | 7081 | 298 | 4.2  (3.7 to 4.7) | 2591 | 162 | 6.3  (5.3 to 7.2) | 7081 | 266 | 3.8  (3.3 to 4.2) | 2591 | 121 | 4.7  (3.9 to 5.5) | 0.5  (0.1 to 0.8) | 0.014 | 1.6  (0.9 to 2.3) | <0.001 |
| Ciatto 2013 | 6079 | 233 | 3.8  (3.4 to 4.3) | 1215 | 80 | 6.6  (5.2 to 8.0) | 6079 | 273 | 4.5  (4.0 to 5.0) | 1215 | 88 | 7.2  (5.8 to 8.7) | -0.7  (-1.1 to -0.2) | 0.003 | -0.7  (-1.9 to 0.5) | 0.285 |
| Zackrisson 2018* | 7705 | 198 | 2.6  (2.2 to 2.9) | 6202 | 338 | 5.4  (4.9 to 6.0) | 7705 | 133 | 1.7  (1.4 to 2.0) | 6202 | 237 | 3.8  (3.3 to 4.3) | 0.8  (0.5 to 1.2) | <0.001 | 1.6  (1.1 to 2.1) | <0.001 |
| Summary estimate | 20865 | 729 | 3.5  (1.9 to 5.2) | 10008 | 580 | 6.1  (4.0 to 8.2) | 20865 | 672 | 3.3  (0.5 to 6.1) | 10008 | 446 | 5.2  (1.1 to 9.4) | 0.2  (-0.6 to 1.1) | 0.600 | 1.0  (-0.1 to 2.1) | 0.070 |
| **US studies** | | | | | | | | | | | | | | | | |
| Alsheik 2019* | 101166 | 7274 | 7.2  (7.0 to 7.3) | 93214 | 9861 | 10.6  (10.4 to 10.8) | 79634 | 7582 | 9.5  (9.3 to 9.7) | 51658 | 6833 | 13.2  (12.9 to 13.5) | -2.3  (-2.6 to -2.1) | <0.001 | -2.6  (-3.0 to -2.3) | <0.001 |
| Conant 2016 | 31493 | 2328 | 7.4  (7.1 to 7.7) | 21133 | 2186 | 10.3  (9.9 to 10.8) | 86103 | 7861 | 9.1  (8.9 to 9.3) | 44303 | 5561 | 12.6  (12.2 to 12.9) | -1.7  (-2.1 to -1.4) | <0.001 | -2.2  (-2.7 to -1.7) | <0.001 |
| Friedewald 2014 | 89171 | 6955 | 7.8  (7.6 to 8.0) | 84243 | 9030 | 10.7  (10.5 to 10.9) | 146910 | 12845 | 8.7  (8.6 to 8.9) | 131996 | 16582 | 12.6  (12.4 to 12.7) | -0.9  (-1.2 to -0.7) | <0.001 | -1.8  (-2.1 to -1.6) | <0.001 |
| Haas 2013 | 3453 | 257 | 7.4  (6.6 to 8.3) | 2639 | 257 | 9.7  (8.6 to 10.9) | 4886 | 485 | 9.9  (9.1 to 10.8) | 2158 | 358 | 16.6  (15.0 to 18.2) | -2.5  (-3.7 to -1.3) | <0.001 | -6.9  (-8.8 to -4.9) | <0.001 |
| McCarthy 2014 | 10515 | 819 | 7.8  (7.3 to 8.3) | 5056 | 547 | 10.8  (10.0 to 11.7) | 7239 | 667 | 9.2  (8.5 to 9.9) | 3489 | 445 | 12.8  (11.6 to 13.9) | -1.4  (-2.3 to -0.6) | 0.001 | -1.9  (-3.3 to -0.5) | 0.007 |
| Rose 2013 | 4833 | 197 | 4.1  (3.5 to 4.6) | 4666 | 321 | 6.9  (6.2 to 7.6) | 6810 | 465 | 6.8  (6.2 to 7.4) | 7009 | 740 | 10.6  (9.8 to 11.3) | -2.8  (-3.6 to -1.9) | <0.001 | -3.7  (-4.7 to -2.7) | <0.001 |
| Sharpe 2016 | 2984 | 172 | 5.8  (4.9 to 6.6) | 2603 | 169 | 6.5  (5.5 to 7.4) | 39110 | 2563 | 6.6  (6.3 to 6.8) | 31063 | 2706 | 8.7  (8.4 to 9.0) | -0.8  (-1.7 to 0.1) | 0.076 | -2.2  (-3.2 to -1.2) | <0.001 |
| Starikov 2015 | 195 | 17 | 8.7  (4.8 to 12.7) | 1875 | 195 | 10.4  (9.0 to 11.8) | 5040 | 716 | 14.2  (13.2 to 15.2) | 7117 | 1416 | 19.9  (19.0 to 20.8) | -5.5  (-9.6 to -1.4) | 0.008 | -9.5  (-11.2 to -7.8) | <0.001 |
| Summary estimate | 243810 | 18019 | 6.9  (5.8 to 7.9) | 215429 | 22566 | 9.5  (8.2 to 10.8) | 375732 | 33184 | 9.2  (7.5 to 10.9) | 278793 | 34641 | 13.3  (10.8 to 15.9) | -1.8  (-2.4 to -1.2) | <0.001 | -3.5  (-4.5 to -2.6) | <0.001 |
| DBT = Digital breast tomosynthesis; DM = Digital mammography; CI = Confidential interval.  * Study authors provided additional data | | | | | | | | | | | | | | | | |

Supplementary figure 5: Sensitivity analysis for difference in recall rate (incremental recall rate) between DBT and DM stratified by breast density (including studies reporting CDR in addition to recall rate)
Breast density was classified as low (BI-RADS a+b) and high (BI-RADS c+d) (see Data extraction). Risk difference is incremental recall rate (DBT-DM), expressed in decimal form. Squares with horizontal lines represent individual study estimates and 95% CIs. Diamonds represent pooled estimates of incremental recall rate for DBT over DM and 95% CIs. Additional data were supplied by study authors for Alsheik et al 2019 and Zackrisson et al 2018. CI = confidence interval; df = degrees of freedom; IV = inverse variance.


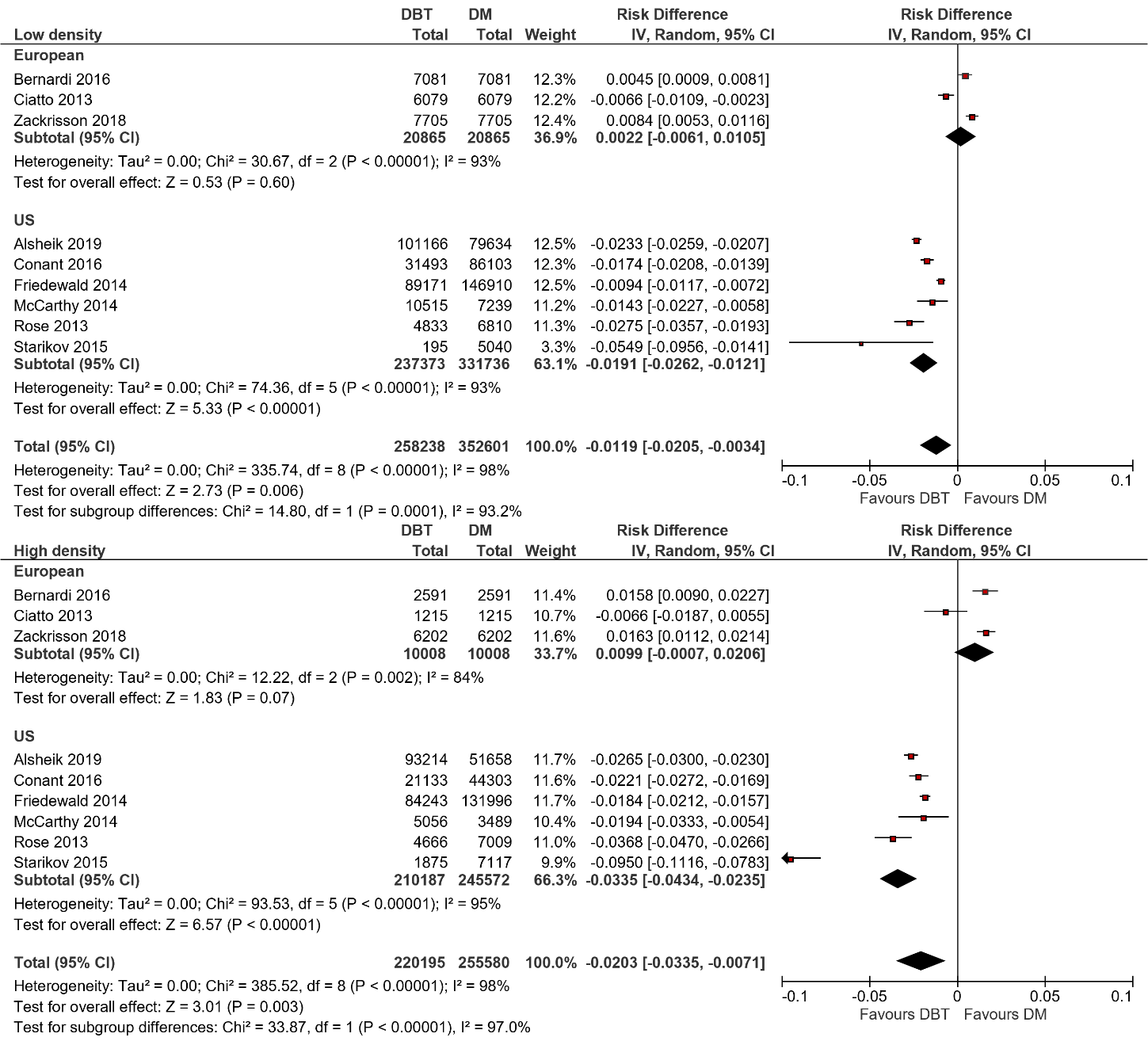


Supplementary figure 6: Sensitivity analysis for difference between high and low density subgroups in DBT’s incremental recall rate (including studies reporting CDR in addition to recall rate)
Breast density was classified as low (BI-RADS a+b) and high (BI-RADS c+d) (see Data extraction). Risk difference is incremental recall rate difference (high-low density), expressed in decimal form. Squares with horizontal lines represent individual study estimates and 95% CIs. Diamonds represent pooled estimates in incremental recall rate for high versus low and 95% CIs. Additional data were supplied by study authors for Alsheik et al 2019 and Zackrisson et al 2018. CI = confidence interval; df = degrees of freedom; IV = inverse variance.


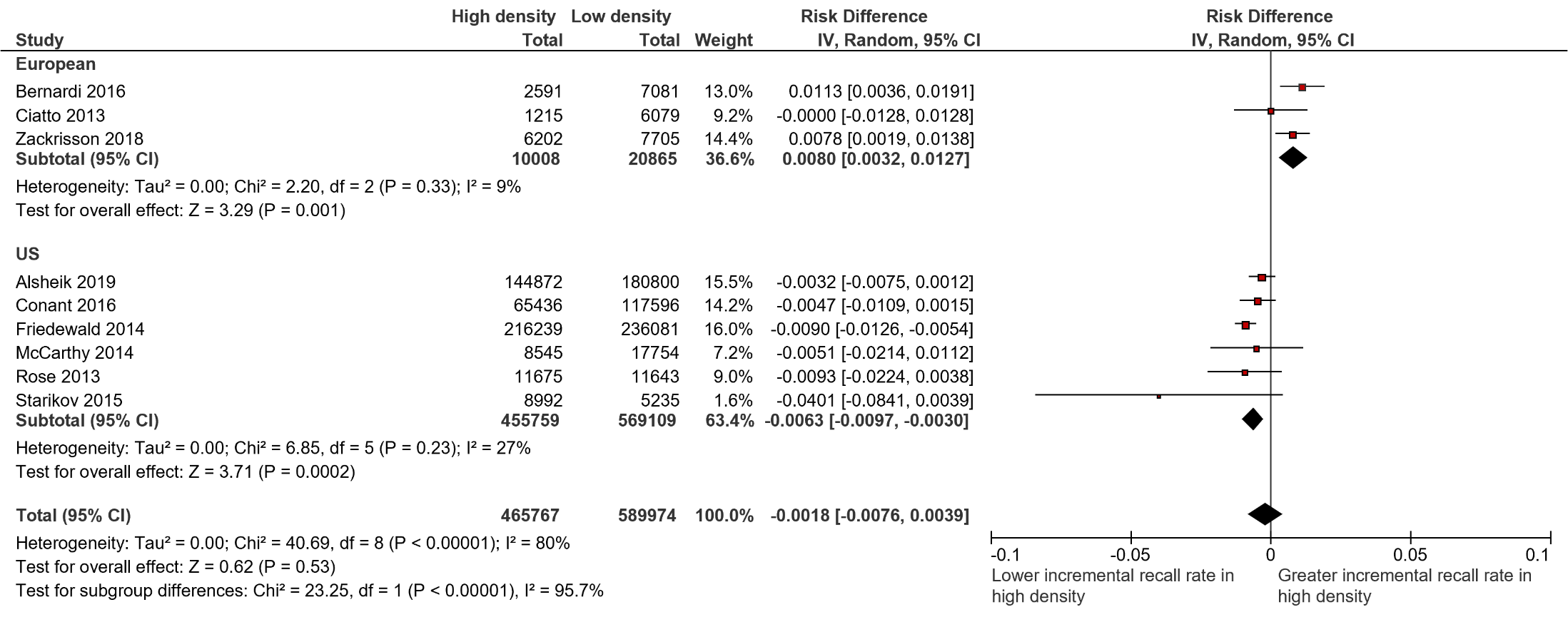


1. In a full decision tree, a *decision node* for the type of test would be followed by *chance nodes* with probabilities for ‘test positive’ (i.e. CDR or recall rate) and ‘test negative’ for both DBT and DM. The additional recalls or cancers detected by DBT would be calculated from the ‘test positive’ *end nodes* for DBT and DM. However, since our meta-analysis considers incremental estimates, this necessitates the simplified decision tree structure presented here where the number of additional cancers and recalls are calculated directly. [↑](#footnote-ref-1)
